# A mini-review on quantification of atherosclerosis in hypercholesterolemic mice

**DOI:** 10.36922/gtm.v1i1.76

**Published:** 2022-06-14

**Authors:** Hui Chen, Deborah A. Howatt, Michael K. Franklin, Naofumi Amioka, Hisashi Sawada, Alan Daugherty, Hong S. Lu

**Affiliations:** 1 Saha Cardiovascular Research Center, University of Kentucky, Lexington, Kentucky, United States; 2 Saha Aortic Center, University of Kentucky, Lexington, Kentucky, United States; 3 Department of Physiology, University of Kentucky, Lexington, Kentucky, United States

**Keywords:** Atherosclerosis, Aorta, En face, Aortic root, Staining, Mouse

## Abstract

Atherosclerosis is a leading cause of morbidity and mortality in many countries. Mice are the most frequently used animal model to study the pathogenesis and molecular mechanisms of atherosclerosis. *En face* analyses of the aorta and cross-sections of the aortic root are the two common modes for quantifying the severity of atherosclerosis in mice. This mini-review introduces these two methods, discusses their pros and cons, and provides suggestions to optimize the quantification of atherosclerosis, thereby enhancing rigor and reproducibility in preclinical research.

## 1. Introduction

Atherosclerosis is a chronic complex pathological process. Two classic mouse strains, apolipoprotein E-null (ApoE^−/−^) mice and low-density lipoprotein receptor-deficient (LDLR^−/−^) mice, have been widely used to study the pathogenesis and molecular mechanisms of atherosclerosis^[1–3]^. Recently, mice manipulated with a proprotein convertase subtilisin/kexin type 9 (*PCSK9*) gain-of-function mutation have become a popular model to study atherosclerosis. Briefly, normocholesterolemic mice are injected with adeno-associated viruses (AAVs) containing either the human D374Y or mouse D377Y gain-of-function mutation of *PCSK9*^[4–8]^. This manipulation leads to LDLR degradation, thereby mimicking the LDLR^−/−^ mouse model. The benefits of using AAVs expressing gain-of-function PCSK9 mutants are two-fold: First, it saves both time and expense for mouse breeding; second, in contrast to constitutive deletion of *LDLR* in LDLR^−/−^ mice, this approach can be performed to delete LDLR in adult mice^[9]^.

In these hypercholesterolemic mouse models, the atherosclerosis-prone locations are the aorta and the major branches of the aortic arch. Quantification of lesion areas in selected vascular beds has been considered a “gold standard” for evaluating the severity of atherosclerosis. This brief review introduces and discusses the two commonly used methods, *en face* analysis in the aortic surface and cross-sections of the aortic root, to quantify atherosclerotic lesion size in mice.

## 2. *En face* analysis of atherosclerotic lesions

**Figure 1.**
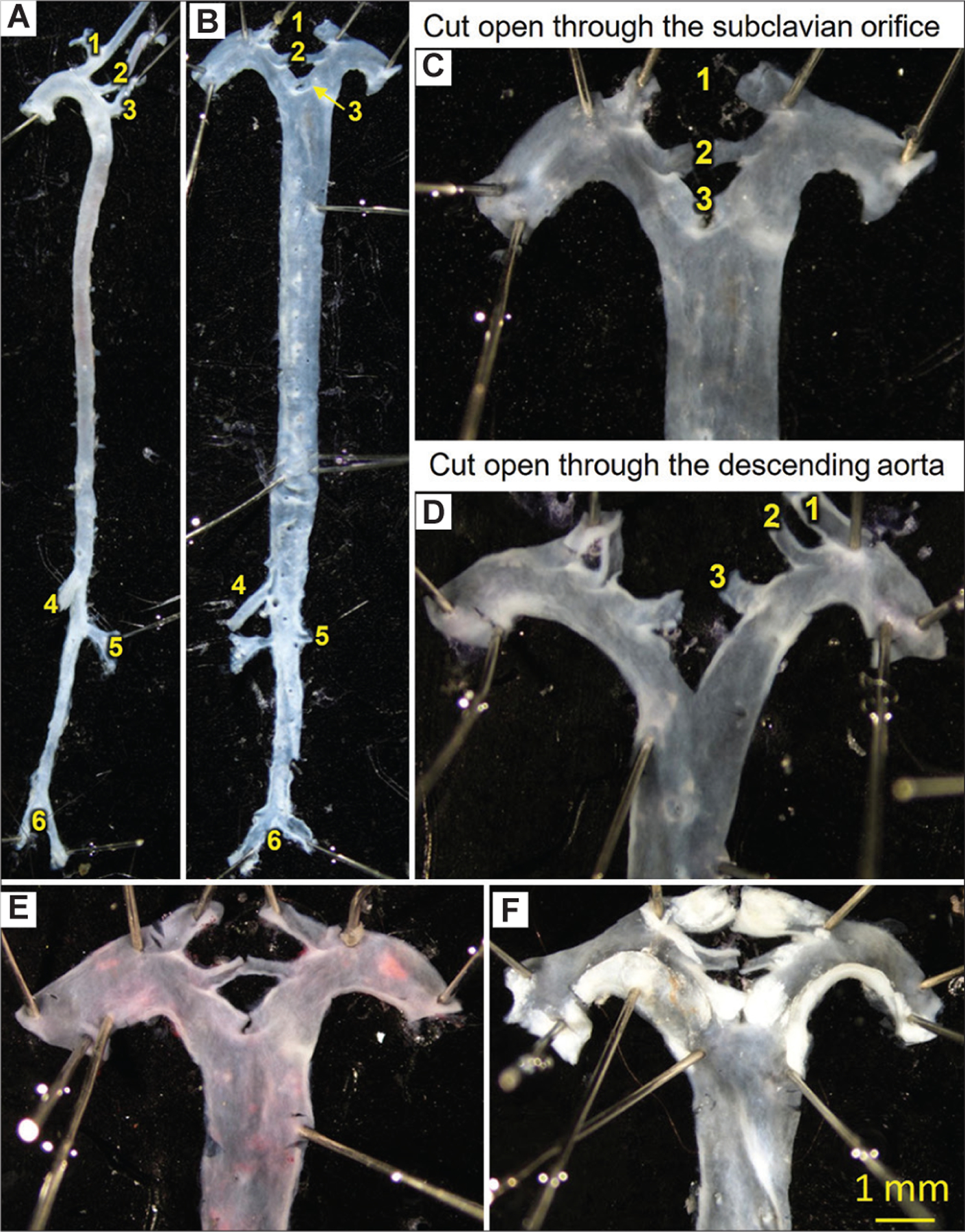
*En face* method for mouse aortas. (A) An example of the normal aorta after cleaning off the adventitia: The important aortic branches including innominate artery, left carotid artery, left subclavian artery, superior mesenteric artery, left renal artery, and iliac bifurcation are remained as landmarks. (B) An example of *en face* aorta from the ascending region to the iliac bifurcation. Comparisons between the two cut-open modes are shown in (C and D). (C) The innominate and left carotid arterial branches are remained, and the aorta is cut open at the orifice of the left subclavian artery. (D) All three branches of the aortic arch are remained and a cut to the outer curvature of the descending thoracic aorta is made. (E) Oil red O staining was performed in an aorta without atherosclerotic lesions. The red color is due to the presence of adipose in the adventitial side. (F) An example of atherosclerotic lesions in the ascending aorta, aortic arch, and the aortic branches without Oil Red O staining. Notes: (1) Innominate artery, (2) left carotid artery, (3) left subclavian artery, (4) superior mesenteric artery, (5) left renal artery, and (6) iliac bifurcation.

*En face* method is used routinely for the entire length of the aorta from the ascending to the abdominal aortic regions above the iliac bifurcation (Figure 1A). After dissection from the mouse, the aorta is fixed in either 10% neutrally buffered formalin or 4% paraformaldehyde for at least 24 h to preserve the tissue. Adventitial tissues are then removed carefully and the aorta is cut open longitudinally through the inner curvature and down the anterior aspect. The aortic arch has three major branches: Innominate artery, left common carotid artery, and left subclavian artery (Figure 1A). These three branches have been used as landmarks to cut open and flat the aorta through the outer curvature. In the published literature, there have been two major modes to cut open the three aortic branches. The first mode is to cut open and retain the innominate and left carotid arteries but cutoff the left subclavian branch, and use its orifice to open the aorta (Figure 1B and C). The second mode is to cut open and retain all three branches, and then make an additional cut of the outer curvature in the descending thoracic aorta (Figure 1D). Although many published articles use the second mode shown in Figure 1D, we recommend using the first method as shown in Figure 1B and C unless researchers want to focus on *en face* analysis of atherosclerosis in the subclavian artery. One shortcoming of the second method as shown in Figure 1D is that it makes an artificial cut in the descending aorta, which is not easy to be consistent unless a landmark is used. This cut may also damage or dislodge lesions in the descending thoracic aorta. In contrast, since the first method as shown in Figure 1B and C makes a cut through the orifice of the subclavian artery, it keeps the consistency and minimizes the damage or displacement of lesions in the descending thoracic aortic region.

Atherosclerotic lesions are most abundant in the ascending aorta, aortic arch, and the major branches of the aortic arch. Therefore, it is important to be extremely careful when cutting open the aorta, which should be performed under a dissecting microscope. The aorta should be immersed in either saline or phosphate-buffered saline to prevent the vessel from drying out. Fine tipped (tip diameter ~ 0.05 mm) Vannas spring scissors should be used for opening the aortic branches due to their small size. We suggest that 1^st^ time users practice using normal aortas and master the technique before performing an atherosclerosis study.

Oil red O staining has been frequently used for *en face* analysis of atherosclerosis. Although the red color enhances the visualization of lesions, it is worth noting that this method also stains neutral lipid of adipose in the adventitia (Figure 1E), which may be mistakenly evaluated as atherosclerotic plaques. Furthermore, some lesions may be fragile and can be displaced during the staining process. This is particularly the case for lesions in mice that have undergone bone marrow transplantation. Considering these issues, there is no tangible benefit to performing staining at least for large and mature plaques (Figure 1F), and in fact, there may be some detriments.

Some software packages provide functions to recognize and automatically measure atherosclerotic lesion areas. However, even with images captured using a high-resolution microscope, it cannot completely avoid lambiguity in the visualization of lesions. On the basis of our long-term experience, we recommend that lesions are measured manually while observing the pinned aorta simultaneously under a dissecting microscope. For *en face* analysis, it is preferable that lesion size be presented as percent lesion area that is normalized by the intimal surface area of the aortic regions measured. This is critical because each aorta has a different surface area; also, it is hard to be uniformly consistent in dissecting and opening aortas.

## 3. Quantification of atherosclerosis using cross-sections of the aortic root

Quantification of atherosclerosis in cross-sections of aortic roots in mice was initially described by Paigen *et al*. in the 1980s^[10]^. This method has become a common approach for quantifying atherosclerosis in mice since then. The recent American Heart Association (AHA) statement recommends cutting serial sections from the aortic valves to the ascending aorta (~8 – 10 μm/section for fresh-frozen tissues embedded in optimal cutting temperature [O.C.T.] compound)^[1]^. Since region-specific differences of lesion size in aortic roots have been noted frequently, the AHA statement suggested to measure lesion area throughout the aortic root^[1]^. Despite lack of standard criteria, we recommend at least measuring lesion areas on five serial sections for each aortic root.

To quantify atherosclerotic lesions in multiple serial sections, it is critical to set up and section aortic roots appropriately. This technique requires the researcher to discern the anatomy of the aortic root that starts from the aortic valves at the left ventricular outlet that locates approximately the top 1/4 of the heart. The heart tissue (the top 1/4) containing the aortic root should be placed in a base mold that is positioned perpendicularly to the bottom surface of the mold and completely covered with O.C.T. Sectioning the tissue starts from the ventricular side. The ventricular tissue is sectioned and discarded until the aortic sinus is reached. This should be identified by frequently checking sections under a microscope until the first two aortic valves appear. We suggest collecting sections once the first two aortic valves appear. Frozen aortic samples should be collected serially at 10 μm per section on ~10 slides until the aortic wall disappears or is not intact anymore. Appropriate setting and successful sectioning should allow the collection of at least nine serial sections per slide for ten slides. We do not recommend sectioning < 8 μm per section for fresh frozen aortic samples due to the fragile nature of the tissue.

**Figure 2.**
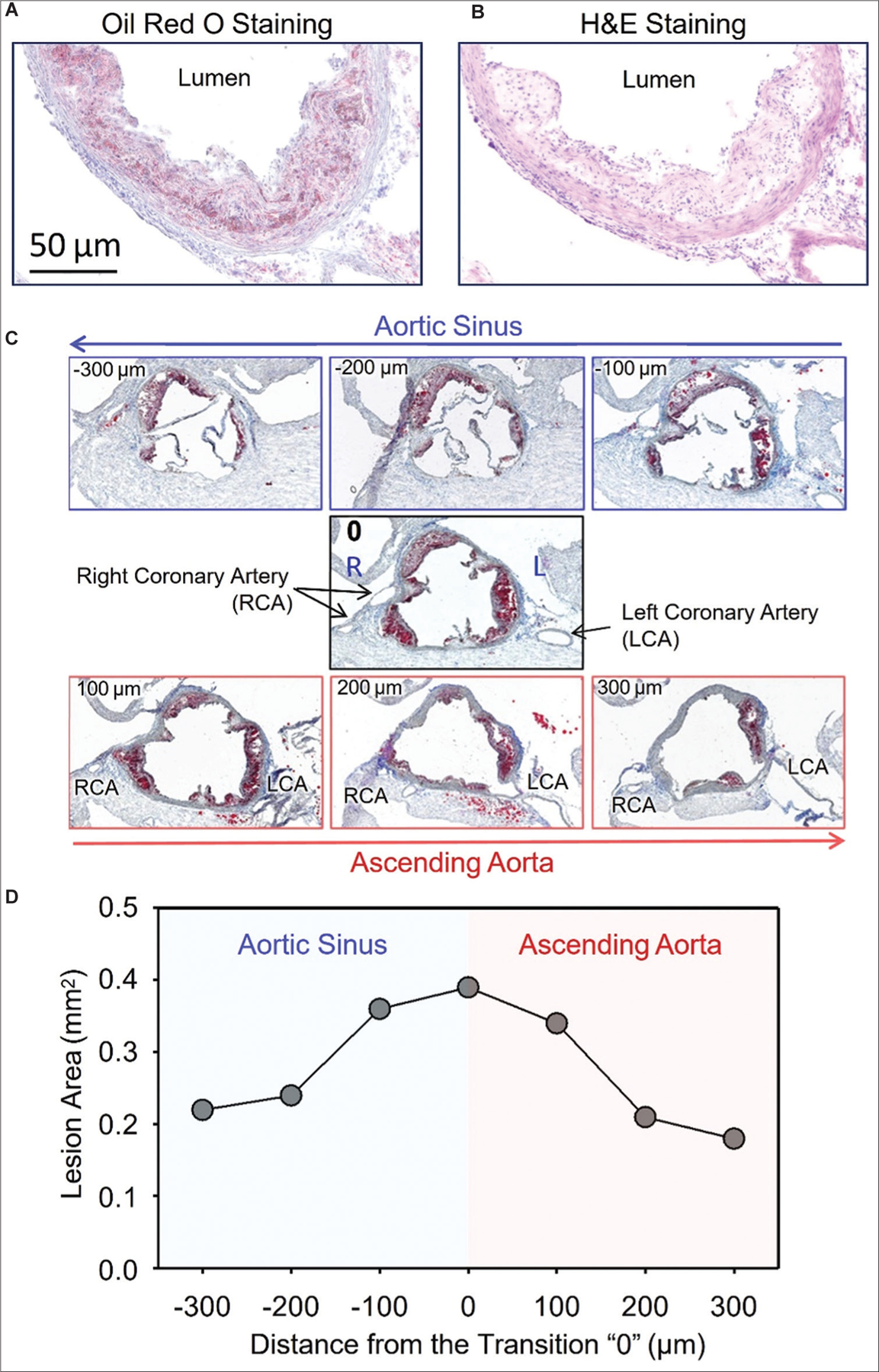
Cross-sections of mouse aortic roots. Comparisons between Oil red O staining (A) and hematoxylin and eosin staining (B) are from two sequential cross-sections of the same aortic root. (C) An example of aortic root serial cross-section images. The distance between the sequential serial sections is 100 µm. (D) An example of presenting lesion areas measured on seven serial sections of an aortic root. Note: RCA, right coronary artery; LCA, left coronary artery.

For cross-sections, lesions can be easily visualized with several types of staining including Oil red O staining, hematoxylin and eosin (H&E) staining, Verhoeff van Geison staining, and Masson trichrome staining. Many works as described in published articles used Oil red O staining (Figure 2A) to visualize lesions since lipid-laden macrophages are the major cells in lesions. However, this staining does not incorporate extracellular matrix, unesterified cholesterol, smooth muscle cells, and some other components. In contrast, H&E staining (Figure 2B) can both visualize the plaques readily and distinguish the lesion boundaries from the internal elastin lamina. It is also an easy and quick staining process. Therefore, we recommend using H&E staining for quantification of lesion areas.

To keep consistency for lesion quantification in the same location for each aortic root, many articles used the presence of the three leaflets of the aortic valve as a landmark. Based on our own experience, this landmark is not easily distinguished because of many variations caused by the anatomical differences and the nature of tissue sectioning. Anatomically, the three leaflets are not on the same level in the heart; technically, it is difficult to set the three aortic leaflets on the same level for cutting. Furthermore, it is hard to appropriately define where the initial appearance of the three leaflets is. Rather than using the initial appearance of the three leaflets, we recommend using the disappearance of the three leaflets as the landmark (“0” in Figure 2C). Although this landmark does not completely overcome the issues mentioned above, it is a more optimal and consistent landmark than the initial appearance of three leaflets. We label the cross-section with disappearance of the three leaflets as the transition point “0,” which represents the ending of the aortic sinus and the beginning of the ascending aorta (“0” in Figure 2C). We routinely measure at least 3–4 serial sections before “0”, and at least 2–4 sections after “0”. Therefore, lesion areas on 6–9 sections (including the “0”) through the aortic root are measured (Figure 2C). Recently, we have also noted that atherosclerotic lesions may be predominant toward a specific location of the aortic valve leaflets. Therefore, we recommend that the presentation of the aortic root sections always follows the same orientation using right and left coronary arteries as the orientation landmarks (Figure 2C).

Plaque areas are quantified using image analysis software. As noted for *en face* analysis, automated quantification of lesion size is not recommended because most staining methods, particularly Oil red O staining, cannot provide uniform staining that covers the entire area of the lesions with comparable intensity. The most accurate method is to manually trace each lesion on cross-sections, although many recent software packages have advanced functions to enhance automated quantification. It is important to note that it is not appropriate to report percent lesion area that is normalized using luminal area for aortic root cross-sections. In contrast to the normalization of lesion area by intimal surface area for the *en face* analysis, luminal area cannot reflect the true surface areas in the aortic root cross-sections. Therefore, most researchers report absolute lesion areas, as we also recommend. Many articles show one representative image for the aortic root, which does not reflect appropriately the lesion measurements. We recommend showing images of the serial sections used for quantification of atherosclerosis, as shown in Figure 2C. While we suggest measuring lesion areas on 6–9 sections of each aortic root, we also recommend reporting the data as per the example shown in Figure 2D.

## 4. Summary

Both *en face* analysis and aortic root cross-section methods have been the classic approaches for atherosclerotic lesion size quantification. In addition to what we have recommended above, it is important to consider some factors for experimental design and data analysis. We recommend taking the following factors into consideration:

- *En face* analysis provides quantification of two-dimensional lesion area without considering the thickness of lesions. To overcome this shortcoming, cholesterol content can be measured, for example, using chromatography. This is particularly insightful if the lesions are thick. Furthermore, we expect in the near future that the high resolution of the advanced micro-CT may make it possible to measure the full three-dimensional volume of plaques in both the aorta and the aortic root.
- Atherosclerotic lesions are initiated in the aortic root, ascending aorta, and arch regions, but hardly detectable in the descending thoracic and abdominal regions within 8–12 weeks of Western diet feeding. We recommend measuring and comparing lesions in a region-specific manner. These will help discover potential region-specific differences.
- In addition to the aorta, atherosclerosis can also be profound in the innominate and left carotid arteries when the mice are fed a Western-type diet for a prolonged duration such as 12 weeks (or 3 months). We recommend including the proximal portions of these two arteries (such as 1 mm from the orifice) for *en face* analysis.
- Evaluation of human and animal atherosclerosis has provided evidence that lesion composition, rather than lesion size, may be the primary factor defining the risk of cardiovascular consequences^[1,11,12]^. In addition to lesion size, it would be important to perform histology and immunostaining to compare lesion composition^[1,13]^.
